# Vastus Lateralis Architecture Changes During Pregnancy – A Longitudinal Study

**DOI:** 10.3389/fphys.2019.01163

**Published:** 2019-09-11

**Authors:** Marie Elena Bey, Robert Marzilger, Larry Hinkson, Adamantios Arampatzis, Kirsten Legerlotz

**Affiliations:** ^1^Department of Training and Movement Sciences, Humboldt-Universität zu Berlin, Berlin, Germany; ^2^Berlin School of Movement Science, Berlin, Germany; ^3^Department of Obstetrics, Charité – Universitätsmedizin Berlin, Berlin, Germany

**Keywords:** muscle architecture, muscle growth, muscle strength, hypertrophy, body composition, exercise, injury, pregnancy

## Abstract

While the incidence of falls has been described to increase with pregnancy, the mechanism behind this is unclear. Pregnancy associated changes in lower extremity muscle strength could be a possible factor influencing injury risk. Thus, the aim of this longitudinal study was to investigate muscle strength and architectural properties of the lower limbs in different stages of pregnancy and postpartum. In nineteen pregnant women (30 ± 4 years) and fifteen non-pregnant controls (28 ± 4 years) muscle strength and architectural properties of the vastus lateralis muscle were assessed combining dynamometry, ultrasound, kinematic, and electromyographic measurements. Body mass and body composition were determined using bioimpedance analysis. In the pregnant women, the measurements were conducted in the 16 ± 4th (EP) and 29 ± 4th week of pregnancy (LP) as well as in the 32 ± 9th week postpartum (PP). Muscle thickness and pennation angle of the fascicles significantly increased at LP, while muscle strength remained constant during and after pregnancy. Body mass, skeletal muscle mass, fat mass, intracellular and extracellular water also peaked at LP. Postpartum values did not differ from the controls. Changes in the muscle properties were not related to changes in body mass and body composition. Conditions during pregnancy promote changes in the vastus lateralis architecture indicating muscle hypertrophy. However, pregnancy did not increase muscle strength while body mass progressively increases. Therefore, in the event of balance perturbations pregnant women may not be able to meet the requirements for the increased physical demand.

## Introduction

Positive effects of exercise during pregnancy are well documented. Thus, regular exercise during pregnancy is recommended to reduce pregnancy associated symptoms such as hypertension or gestational diabetes, as well as to maintain muscle strength and endurance to prepare for delivery (Vladutiu et al., 2010; Nascimento et al., 2012). However, the safety of exercise during pregnancy has been questioned, as pregnant women are predisposed to falls (Dunning et al., 2003; Inanir et al., 2014). The incidence of falls in pregnant women has been reported to be 27%, which is similar to the incidence of falls occurring in the elderly (Dunning et al., 2010). Physical changes during pregnancy such as weight gain and the changed body shape have been assumed to influence the risk of falling. In addition, pregnant women have repeatedly been observed to suffer from reduced static and dynamic postural stability compared to non-pregnant women (Oliveira et al., 2009; Inanir et al., 2014; Bey et al., 2018). These impairments in stability are assumed to contribute to the increased number of falls (Inanir et al., 2014).

Hormonal changes during pregnancy are reported to partially account for the decline in stability. The increased level of relaxin for example, has been described to increase the compliance of the pelvic ligaments (Young, 1949) which may lead to joint instability.

Changes in the properties of peripheral skeletal muscles may also affect postural stability and injury risk. A loss in muscle strength, especially of the lower extremities, has been found to be associated with the incidence of falls in the elderly (Moreland et al., 2004). In pregnant women, the evidence for changes in the peripheral skeletal muscles is rare, since most studies mainly focused on muscle strength of the pelvic floor muscles and abdominal muscles due to the increased risk of incontinence during pregnancy (Morkved et al., 2004; Smith et al., 2007; Gameiro et al., 2011). To our knowledge, there are only two studies investigating changes in strength of the upper and lower extremities with pregnancy. One study investigated changes in the hand grip strength in pregnant women, detecting a 9% loss in strength in the late stage of pregnancy compared to the middle stage of pregnancy (Atay and Basalan Iz, 2015). Another study conducted measurements of the lower and upper body strength before pregnancy and 6 weeks postpartum (Treuth et al., 2005). These authors also established a loss in strength, with the largest loss occurring in the lower body with 24%. However, the authors did not include measurements during pregnancy. Therefore, the results are more likely to reflect the changes during the recovery phase after childbirth than changes during pregnancy. The described loss in muscle strength in pregnant women may be related to the progressive increase in fat mass (Taggart et al., 1967). An increase in fat mass is known to reduce the desire for spontaneous physical activity (Brown, 2008), which, in turn, leads to muscle weakness (Atay and Basalan Iz, 2015).

Although these studies suggest that pregnancy is associated with muscle weakness, hormonal changes during pregnancy can also lead to muscle growth as it has been shown for the uterus muscle (Rundgren, 1974). Since pregnancy has been shown to improve the regenerative processes in skeletal muscles (Falick Michaeli et al., 2015), skeletal muscles of pregnant women may respond well to anabolic stimuli such as increased loading. As an increased body mass has been found to increase the anatomical cross-sectional area of the quadriceps femoris muscle (Delmonico et al., 2009), the progressive increase in body mass during pregnancy may potentiate the effect to trigger radial muscle growth in the lower extremities. Muscle strength during pregnancy may also be affected by water retention, which is a common side effect occurring with pregnancy (Lukaski et al., 1994; Valensise et al., 2000). While we know that total body water content affects muscular performance with a reduction in total body water being associated with a reduction in muscle strength (Judelson et al., 2007), it has not yet been investigated how or if muscle performance in pregnant women is affected by pregnancy associated water retention.

In the event of pregnancy associated reductions in muscle strength these would likely affect the injury risk during exercise as well as during daily activities. However, the lack of studies investigating pregnancy related changes in peripheral skeletal muscle properties does not allow drawing clear conclusions. To be able to develop effective and safe interventions for pregnant women, similar to the strategies for addressing muscle strength and postural stability to reduce the risk of falls in the elderly (Moreland et al., 2004), the effect of pregnancy on skeletal muscles needs to be clarified.

Therefore, the aim of this longitudinal study was to investigate muscle properties of the knee extensors at two stages of pregnancy and 6 months after delivery.

We hypothesized that knee extensor muscle strength, thickness, and pennation angle will increase at the late stage of pregnancy due to the increased body mass. We hypothesized furthermore, that with a reduction in body mass in the postpartum period values will return to pre-pregnancy levels and not be significantly different from the non-pregnant controls. In addition, increases in muscle thickness may be affected by water retention. Thus, we further hypothesized that changes in muscle properties are related to changes in body mass and body composition.

## Materials and Methods

### Participants

Muscle properties, body mass and body composition during and after pregnancy were analyzed longitudinally in nineteen pregnant women (30 ± 4 years). Due to the lack of longitudinal data on changes in muscle properties in pregnant women we were not able to conduct an *a priori* power analysis to determine the sample size. However, previous studies in our department have shown that this sample size is sufficient to detect training induced changes in the vastus lateralis muscle such as significant increases in muscle strength (8%, *p* = 0.003) and thickness (24%, *p* < 0.001) (Mersmann et al., 2016). All women were healthy without any orthopedic or pregnancy associated disorders. Pregnant women with a multiple pregnancy were excluded from the study. The participants attended three experimental sessions in different stages of pregnancy: the first measurement was conducted in early pregnancy [EP, 16 ± 4 week of pregnancy (WoP)], the second measurement in the late stage of pregnancy (LP, 29 ± 4 WoP) and the third measurement at least 6 months postpartum (PP, 32 ± 9 weeks after delivery) to reflect the non-pregnant status. In two of the pregnant women an additional measurement had been conducted prior pregnancy.

In order to compare the postpartum status of the pregnant women with data of non-pregnant controls an additional cohort of fifteen healthy non-pregnant women (28 ± 4 years) was recruited. For the non-pregnant group, the same data were collected as for the pregnant group, while the measurements in the non-pregnant group were conducted once.

Within this research project further data such as tendon properties were obtained and published as separate article (Bey et al., 2019).

### Measurement of Muscle Properties

To assess the muscle strength of the knee extensors and the architecture of the vastus lateralis muscle the women were seated on a dynamometer (BIODEX Medical System 3, Shirley, NY, United States). In order to monitor the knee joint angle and to precisely calculate the knee extensor moment (Arampatzis et al., 2004) kinematic measures were performed using seven Vicon cameras (VICON Motion Systems, version 1.7.1, Oxford, United Kingdom) at a frame rate of 250 Hz.

For data capturing five reflective markers were placed at the trochanter major, the lateral and medial epicondyle of the femur as well as the lateral and medial malleolus of the dominant leg. Standing barefoot in an upright body position with straight legs the vertical connection between the trochanter major and the lateral epicondyle was marked on the skin using a non-permanent marker. The length of the line was defined as the length of the femur. After marking the half of the femur length with a horizontal line the thigh perimeter was measured directly above the intersection of both lines. Subsequently, the participants took a seat on the dynamometer with an 85° trunk angle.

#### Muscle Strength

To examine muscle strength the women completed five trials of slow isometric ramp contractions with a 90° knee joint angle. Muscle strength was assessed determining the knee joint moment on the dynamometer. Gravitational forces as well as a misalignment between the knee joint and the dynamometer axis during the contractions have been reported to lead to overestimation of the knee joint moment of up to 17% (Arampatzis et al., 2004). In order to consider this we conducted simultaneous measurements of kinematic and dynamometric data during the ramp contractions. The corrected knee joint moment was calculated through inverse dynamics (Arampatzis et al., 2004). Furthermore, since the contribution of the knee flexors has been found to affect the knee extensor moment by 6% (Mademli et al., 2004), we additionally subtracted the knee flexor moment from the corrected knee joint moment. The knee flexor moment was assessed by electromyographic based estimates of the knee flexors’ co-activation (Mademli et al., 2004). Details of the correction procedures have been described in previous studies (Arampatzis et al., 2004; Mademli et al., 2004; Bey et al., 2019).

The relative knee joint moment was calculated normalizing the absolute knee joint moment to the body mass. In two women of the control group we were not able to analyze the muscle strength due to an inadequate recording of the knee joint moment.

#### Architecture of Vastus Lateralis Muscle

To assess architectural properties of the vastus lateralis muscle the knee joint was flexed to 60°. A 10 cm ultrasound transducer (7.5 MHz, My Lab60, Esaote, Genova, Italy) was positioned longitudinally to the muscle. A custom made plastic cast around the transducer with a ∼2.5 cm width on the long sides prohibited a possible tilt of the probe during measurement. One long side of the cast was positioned on the connecting line between trochanter major and lateral epicondyle with the middle of the transducer being placed at the middle of the femur. In this position, two ultrasound videos were captured at 25 Hz while the participants were instructed to keep their leg muscles relaxed.

In ten consecutive frames of each video the upper and deeper aponeurosis were manually traced using a custom written Matlab interface (Marzilger et al., 2018). Muscle thickness was determined calculating the distance between the aponeuroses. Visible snippets of inter-fascicular collagen were semi-automatically detected by the program, and a reference fascicle was generated based on the different characteristics of the snippets. The pennation angle and fascicle length were calculated from the reference fascicle in respect to the aponeuroses. Fascicle length in the pregnant women was reported as absolute values. To compare the pregnant to the non-pregnant group, fascicle length was normalized to the femur length (FL_norm_) to account for differences in body height (Table 1) between these groups.

**TABLE 1:** Anthropometric data for the non-pregnant controls and the pregnant women at the early (EP) and late (LP) stage of pregnancy as well as postpartum (PP) (means ± standard deviation).

**Groups**	**Week**	**Age (year)**	**Body height (cm)**	**Body mass index (kg/m^2^)**	**Thigh perimeter (cm)**
Controls	–	27.8 ± 4.1	166 ± 6	23.4 ± 2.7	53.0 ± 3.9
EP	16 ± 4 WoP	30.1 ± 4.3	170 ± 6^#^	23.0 ± 2.9	51.5 ± 2.9
LP	29 ± 4 WoP	30.3 ± 4.3	–	25.1 ± 3.3^∗^	52.9 ± 2.9^∗^
PP	32 ± 9 after delivery	31.2 ± 4.2^†#^	–	22.6 ± 4.0	50.5 ± 3.3

*^†^significantly different to EP and LP (*p* < 0.05). ^∗^significantly different to EP and PP (*p* < 0.05). ^#^significantly different to the controls (*p* < 0.05).*

### Bioelectrical Impedance Measurement

To assess the body composition a bioelectrical impedance analysis was performed using the InBody 720 (Biospace Co., Korea). Measurements were conducted according to the manufacturer’s guidelines. Immediately prior stepping on the scale the hand and foot electrodes were cleaned with an antibacterial tissue. Subsequently, the participants stood barefoot and lightly dressed on the scale. Each foot was placed on one heel and one forefoot electrode. The participants were instructed to grab the handles and to touch the two hand electrodes with the thumb and the four fingers. During the measurement the participants stood motionless with a straight body posture and the arms slightly abducted. Impedance values were produced from six different frequencies, from which the resistance of the trunk, the arms, and the legs was calculated. To assess changes in body composition the components body mass (BM), skeletal muscle mass (SMM), and fat mass (FM) in kilogram as well as the total body water (TBW), the intracellular (ICW) and extracellular water (ECW) in liter were analyzed. Accuracy and test-retest reliability for body composition estimations have previously been reported (Lukaski et al., 1986; Lukaski and Bolonchuk, 1988; Kim and Kim, 2013; Legerlotz et al., 2018).

### Statistical Analysis

Normality of the standardized residuals of all investigated parameters were tested in SPSS (Version 21, 32 Bit, IBM, United States) using the Shapiro-Wilk test. A one-way repeated measures ANOVA was performed to analyze differences between the time-points EP, LP, and PP, thereby considering the assumption of sphericity. In case of violations of sphericity the Greenhouse-Geisser correction was used. For *post hoc* comparisons between the measurement time points EP, LP, and PP paired *t*-tests with Bonferroni adjustment were performed. For pairwise comparisons of the not normally distributed parameters (BM at PP, BMI at PP, fascicle length at LP and PP, thickness at PP, pennation angle at EP as well as FM at LP and PP) the Friedman’s test and the Wilcoxon signed-rank test were conducted.

Differences between the postpartum measures and the non-pregnant controls were analyzed using an independent samples *t*-test. The not normally distributed parameters (BMI, SMM, FM, TBW, and ECW for the controls and the above mentioned not normally distributed parameters for the pregnant group) were tested with the Mann-Whitney *U* test. For the normally distributed data the effect size was assessed using Cohen’s d. For the not normally distributed data the effect size r was calculated dividing the *z*-scores of the non-parametric tests by the square root of the number of total observations. Thereafter, *r* was converted into *d*.

To investigate the relationship between the muscle properties and body composition parameters we analyzed the Pearson correlation coefficients. The level of significance was set at α = 0.05.

## Results

### Anthropometric Measures

**FIGURE 1:**
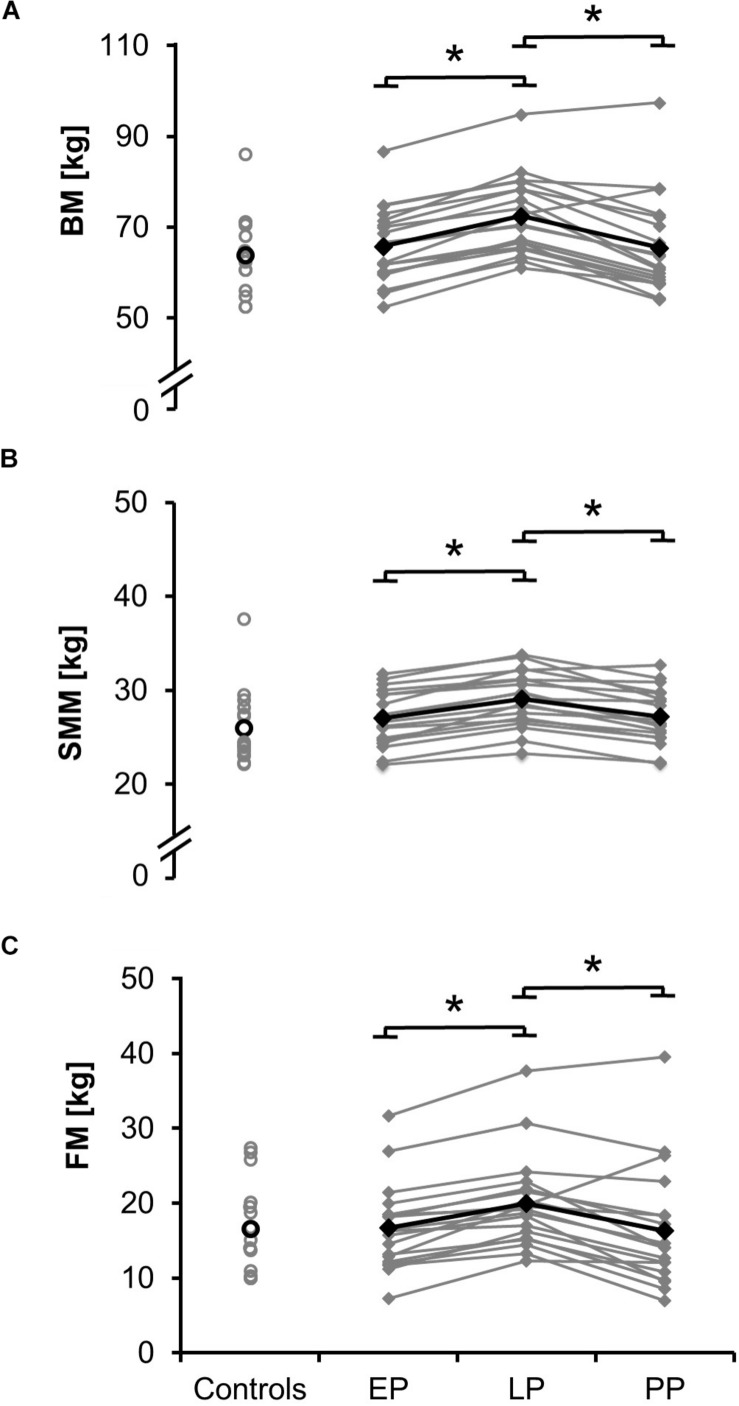
Means (black) and individual data (gray) for body mass BM **(A)**, the skeletal muscle mass SMM **(B)**, and fat mass FM **(C)** for the non-pregnant controls and the pregnant women at the early (EP) and late (LP) stage of pregnancy as well as postpartum (PP) (^∗^*p* < 0.05).

As expected, body mass (Figure 1A), body mass index (BMI), and the thigh perimeter (Table 1) significantly increased from early to late pregnancy (d_mass_ = 3.29, *p* < 0.001; d_BMI_ = 3.65, *p* < 0.001; d_perimeter_ = 0.485, *p* < 0.001) and significantly dropped after delivery (d_mass_ = 2.88, *p* < 0.001; d_BMI_ = 2.88, *p* < 0.001; d_perimeter_ = 0.746, *p* < 0.001). Body mass, BMI, and the thigh perimeter in PP were not different from the non-pregnant controls (*p* > 0.05). Body height and age in the pregnant women (Table 1) were significantly higher compared to the non-pregnant women (d_height_ = 0.72, *p* = 0.045; d_age_ = 0.79, *p* = 0.03).

### Muscle Strength

Knee extensor muscle strength, represented by the absolute knee joint moment, did not change during or after pregnancy (EP: 144.0 ± 34.8 Nm, LP: 146.9 ± 37.1 Nm, PP: 140.6 ± 33.9 Nm), while the relative knee joint moment, normalized to body mass, was significantly smaller at LP (2.07 ± 0.60 Nm/kg, *d* = 1.44, *p* = 0.029) compared to EP (2.21 ± 0.60 Nm/kg) and PP (2.21 ± 0.60 Nm/kg). The postpartum absolute and relative knee extensor moments were not significantly different (*p* > 0.05) from the controls (Moment_abs_ = 145.2 ± 31.8 Nm, Moment_rel_ = 2.36 ± 0.55 Nm/kg).

### Vastus Lateralis Architecture

**FIGURE 2:**
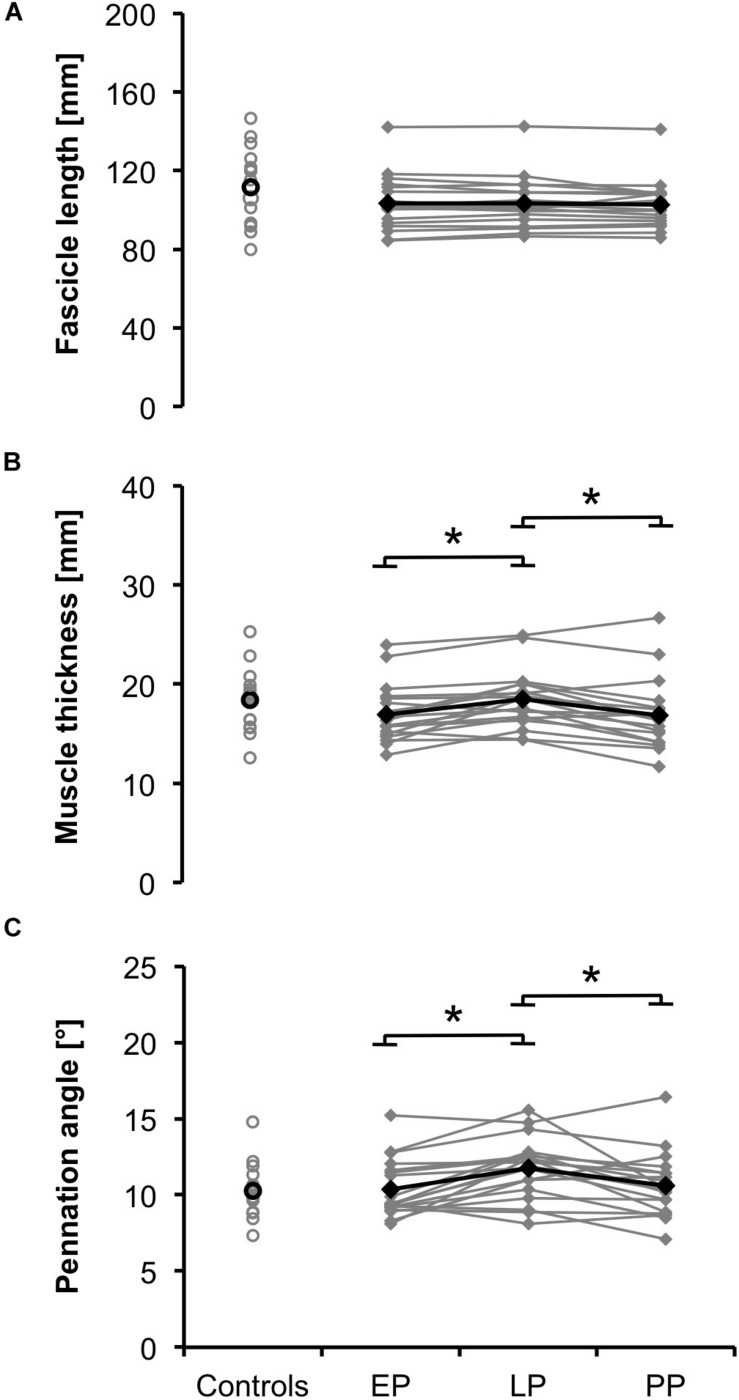
Means (black) and individual data (gray) for the architectural parameters fascicle length **(A)**, muscle thickness **(B)**, and pennation angle **(C)** for the non-pregnant controls and the pregnant women at the early (EP) and late (LP) stage of pregnancy as well as postpartum (PP) (^∗^*p* < 0.05).

Muscle thickness and pennation angle significantly increased during pregnancy (d_thickness_ = 2.19, *p* = 0.001; d_angle_ = 1.97, *p* = 0.002) and significantly dropped postpartum (d_thickness_ = 2.13, *p* = 0.001; d_angle_ = 1.47, *p* = 0.01) (Figures 2B,C). Fascicle length remained constant during and after pregnancy (Figure 2A). The postpartum architectural parameters were not significantly different from the non-pregnant controls (FL_norm, PP_ = 0.28 ± 0.05; FL_norm, Controls_ = 0.32 ± 0.07, *p* > 0.05).

### Body Composition

**FIGURE 3:**
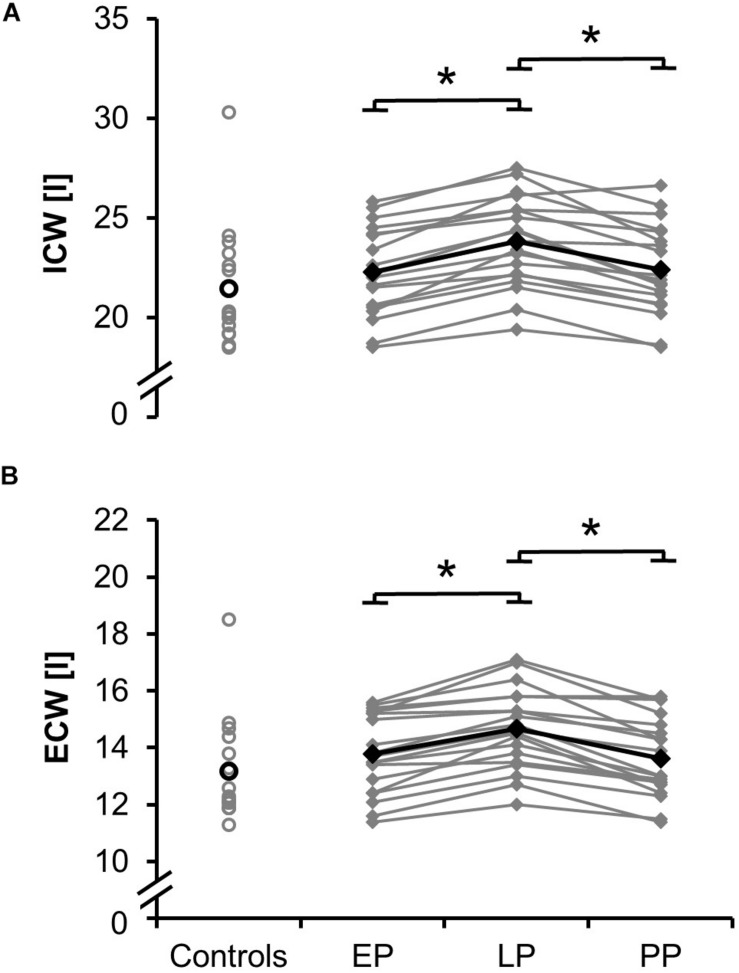
Means (black) and individual data (gray) for the intracellular water ICW **(A)** and extracellular water ECW **(B)** for the non-pregnant controls and the pregnant women at the early (EP) and late (LP) stage of pregnancy as well as postpartum (PP) (^∗^*p* < 0.05).

Total values for skeletal muscle mass (Figure 1B; relative proportion: EP: 41.0 ± 3.0%, LP: 40.4 ± 3.2%, PP: 42.2 ± 4.3%), fat mass (Figure 1C; relative proportion: EP: 24.8 ± 6.0%, LP: 27.2 ± 5.3%, PP: 24.2 ± 7.4%), total body water (absolute proportion: EP: 36.1 ± 3.5 l, LP: 38.5 ± 3.5 l, PP: 36.0 ± 3.6 l; relative proportion: EP: 54.8 ± 3.9%, LP: 53.5 ± 4.2%, PP: 55.9 ± 5.8%), intracellular and extracellular water (Figures 3A,B) significantly increased during pregnancy (d_SMM_ = 0.70, *p* < 0.001; d_FM_ = 3.61, *p* < 0.001; d_TBW_ = 0.69, *p* < 0.001; d_ICW_ = 0.10, *p* < 0.001; d_ECW_ = 0.64, *p* < 0.001) while they significantly dropped postpartum (d_SMM_ = 0.62, *p* < 0.001; d_FM_ = 1.87, *p* = 0.003; d_TBW_ = 0.70, *p* < 0.001; d_ICW_ = 0.64, *p* < 0.001; d_ECW_ = 0.79, *p* < 0.001). Body composition (relative proportion: SMM: 40.8 ± 4.4%, FM: 25.6 ± 7.4%, TBW: 54.5 ± 5.5%) of the non-pregnant controls did not differ from the postpartum measurements.

### Relationships Between Muscle Architecture, Body Mass and Water Content

**FIGURE 4:**
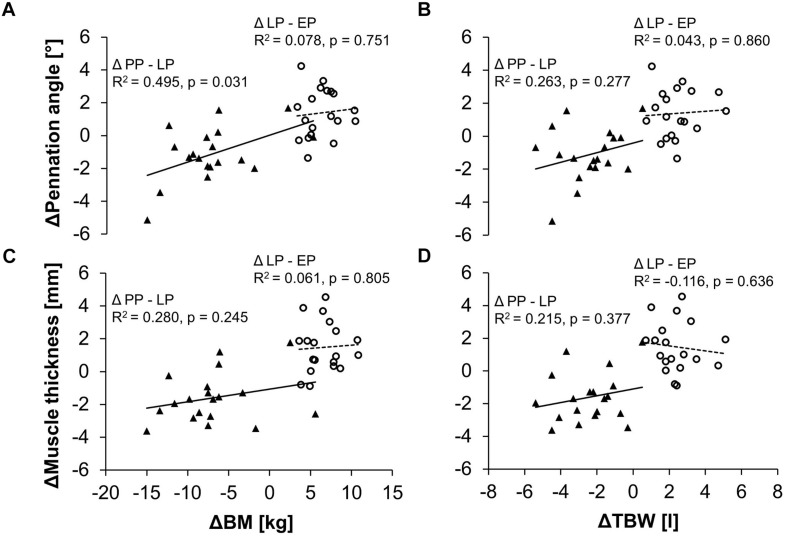
Relationship between the changes in body mass (BM) and the changes in the pennation angle **(A)** or muscle thickness **(C)** as well as between the changes in the total body water (TBW) and the changes in the pennation angle **(B)** or muscle thickness **(D)** in the pregnant women. Demonstrated are the differences of the parameters between the early and late stage of pregnancy (Δ LP-EP, circles) as well as between the postpartum period and the late stage of pregnancy (Δ PP-LP, triangles) and the respective linear regression lines (dashed line for Δ LP-EP, solid line for Δ PP-LP).

Changes in the pennation angle from late pregnancy (LP) to after delivery (PP) correlated moderately with changes in body mass (Figure 4A). The larger the decrease of body mass between LP and PP the larger the decrease in the pennation angle. Changes in body mass during pregnancy (between LP and EP) did not correlate with changes in pennation angle. No significant correlations were found for the relationships between the changes in the body mass and the changes in muscle thickness (Figure 4C) as well as between the changes in the total body water and changes in the pennation angle (Figure 4B) or muscle thickness (Figure 4D).

Changes in skeletal muscle mass from early to late pregnancy and late pregnancy to the postpartum period correlated significantly with changes in the intracellular and extracellular water (EP → LP: ICW: *r* = 0.920, *p* < 0.001; ECW: *r* = 0.823, *p* < 0.001; LP → PP: ICW: *r* = 0.962, *p* < 0.001; ECW: *r* = 0.831, *p* < 0.001).

### Individual Data Sets

In addition to the measurement time-points EP, LP, and PP we were able to measure two women additionally 37 and 36 weeks prior pregnancy (Table 2). Similar to the group mean values, pennation angle, muscle thickness, body content, and water distribution parameters increased at the late stage of pregnancy while the absolute knee joint moment remained constant. After delivery the body content and water distribution returned to the prior pregnancy levels.

**TABLE 2:** Changes in the vastus lateralis muscle architecture, body composition, and water distribution for two women (Women-A: age 34 years, height 169 cm; Women-B: age 26 years, height 162 cm) for the four measurement time-points prior pregnancy, in the early (EP) and late (LP) stage of pregnancy as well as postpartum (PP).

**Session**	**Week**	**Knee extensor moment (Nm)**	**Fascicle length (mm)**	**Muscle thickness (mm)**	**Pennation angle (°)**	**BM (kg)**	**SMM (kg)**	**FM (kg)**	**ICW (l)**	**ECW (l)**
**Woman-A**										
prior	37 pre	144.7	79.6	12.6	10.5	56.2	22.3	15.1	18.6	11.3
EP	12 WoP	159.2	84.4	12.8	9.4	55.5	22.1	14.5	18.5	11.4
LP	28 WoP	162.0	86.4	15.3	11.9	63.6	23.3	20.4	19.4	12.0
PP	31 post	156.8	85.8	13.8	10.3	57.4	22.3	16.1	18.6	11.5
**Woman-B**										
prior	36 pre	175.5	92.6	12.9	12.2	54.8	24.5	10.1	20.3	12.3
EP	14 WoP	162.3	95.3	18.5	11.5	52.4	24.9	7.3	20.6	12.4
LP	27 WoP	164.5	97.7	18.7	12.4	61.0	27.0	12.3	22.2	13.4
PP	25 post	147.1	95.7	17.5	10.9	57.7	24.9	12.1	20.6	12.8

## Discussion

This longitudinal study investigated the effect of pregnancy on muscle properties of the lower extremities measured at the early and late stage of pregnancy as well as 6 months after delivery. While knee extensor muscle strength remained constant during and after pregnancy we found an increase in muscle thickness and pennation angle of the vastus lateralis in the late stage of pregnancy returning to non-pregnant levels in the postpartum phase. Thus, regarding knee extensor muscle thickness and pennation angle our first hypothesis is confirmed, while regarding muscle strength we have to dismiss our hypothesis. Alterations in muscle properties were neither directly associated with changes in the body mass nor with changes in the body composition. Thus, our second hypothesis had to be rejected.

The pregnancy induced change in muscle architecture may be attributed to a combination of changes in the endocrine system, body mass, and body composition. As there are no comparable studies investigating the effect of those factors on architectural properties of peripheral skeletal muscles in pregnant women three probable factors influencing the structure of the muscle are discussed separately: (1) loading, (2) water content, and (3) hormone levels during pregnancy.

An increase in muscle thickness and pennation angle is usually related to the formation of new sarcomeres in parallel (Reeves et al., 2006). This protein based alteration in the muscle structure is known as an adaptive response to overload, e.g., through exercise (Aagaard et al., 2001), or to an increased functional demand, e.g., through a surgical removal of a synergistic muscle (Johnson and Klueber, 1991). Also increases in body mass have been observed to trigger alterations in the quadriceps femoris muscle with an increase in the anatomical cross-sectional area (Delmonico et al., 2009). We may argue that the 10% increase in body mass (6.6 ± 2.1 kg, range: 3.6–10.8 kg) from the 16th to the 29th week of pregnancy may have promoted hypertrophy of the vastus lateralis muscle, thereby increasing the skeletal muscle mass and the thigh perimeter. However, we may question that weight gain during pregnancy is the single factor contributing to alterations in muscle architecture which is somehow in agreement with our results demonstrating that neither the changes in muscle thickness nor the changes in pennation angle from the early to the late stage of pregnancy significantly correlated with the changes in body mass. It is also conceivable that the increase in muscle thickness may have been influenced at least partly by changes in water content during pregnancy, since we detected a significant increase of the intracellular and extracellular water at the late stage of pregnancy.

A progressive increase in the water content during pregnancy has been reported by numerous studies (Lukaski et al., 1994; Valensise et al., 2000; Larciprete et al., 2003) and has been suggested to be fundamental for the expansion of the plasma volume, facilitating the increase in the mother’s cardiac output (Lukaski et al., 1994; Valensise et al., 2000; Larciprete et al., 2003; Cho et al., 2011). After delivery, plasma volume returns to the non-pregnant levels (Hytten and Paintin, 1963), and the water content decreases again (Lukaski et al., 1994; Cho et al., 2011). This reduction in the water content can also be observed in our postpartum data. Since edema in the lower limbs are known to increase the circumference of the leg (Berard et al., 2002), it seems conclusive that water retention during pregnancy may have partly contributed to the 1.9 cm increase in the thigh perimeter in the late stage of pregnancy. However, the increased perimeter cannot solely be attributed to edema as this mainly occurs in the extracellular space. The increase in the intracellular water content could also have led to a thickening of the muscle fibers which could have resulted in a measureable thickening of the muscle tissue. However, it seems that water retention within the muscles does not affect muscle architecture since body water and muscle thickness were not related to each other. We need to point out that we were not able to subtract the content of amniotic fluid from the total body water, which may have affected our results.

Hormonal changes during pregnancy could also have affected muscle architecture. The endocrine system is known to modulate anabolic processes in muscles and to affect muscle plasticity (Hackney, 1999; Hansen and Kjaer, 2014). While increased estrogen levels during pregnancy have been associated with uterus muscle growth (Rundgren, 1974), the anabolic effect of estrogen on peripheral skeletal muscles is still debated (Hansen et al., 2003). However, as observed in the hind limbs in mice gestation seems to improve the regeneration processes in skeletal muscles and to counteract negative effects of aging (Falick Michaeli et al., 2015). This improvement in regenerative capabilities during pregnancy may hint toward favorable metabolic conditions promoting the adaptation to training and mechanical loading.

While our results clearly indicate, that pregnancy leads to changes in muscle architecture, and that this change is not caused by a single factor, it remains to be established which pregnancy associated combination of factors contributes to this change. Furthermore, it needs to be established how this change in muscle architecture affects muscle function.

Despite increases in muscle thickness we did not observe any increase in the knee extensor muscle strength. To our knowledge, no study has yet investigated lower limb muscles during pregnancy. A study on upper limb muscles has observed a 9% decline in handgrip strength in the late stage of pregnancy compared to the middle stage of pregnancy (Atay and Basalan Iz, 2015). It is possible that both the upper and lower body are subjected to different adaptation processes during pregnancy. Regional differences in muscle adaptation have previously been observed in other scenarios (Bemben et al., 1991; Janssen et al., 2000). As requirements regarding balance maintenance and stabilization of the heavier body during standing and locomotion are enhanced during pregnancy, a reduction in lower body strength seems unreasonable from an evolutionary perspective. Changes in the vastus lateralis muscle architecture may point toward an adaptive response to meet the increased demands. However, absolute muscle strength did not increase. Muscle strength relative to body mass even dropped in late pregnancy. As consequence, it seems likely that in the event of balance perturbations muscle strength may not be sufficiently high enough to prevent the fall of a heavier body.

The dimension of force production in the late stage of pregnancy may also be affected by changes in the women’s fitness level during pregnancy. Atay and Basalan Iz (2015) state that the loss in upper limb muscle strength in pregnant women is primarily attributed to a reduction in physical activity. However, even if our participants may have progressively reduced the intensity of their activities during pregnancy this did at least not lead to a loss in the knee extensor muscle strength. Further, psychological factors may have affected force production during the late stage of pregnancy. Primarily in the 29th week of pregnancy, when the fetus is getting close to its birth length, our participants may not have felt confident to produce the maximum isometric force on the dynamometer. In addition, elevated anxiety levels may have influenced the women’s performance during the ramp contraction trials. As the prevalence of self-reported anxiety has been reported to continuously increase during pregnancy from 18% in the first trimester to 25% in the third trimester, while decreasing in the postpartum period (Dennis et al., 2017; Nakic Rados et al., 2018), the women may have unconsciously changed their behavior in the late stage of pregnancy.

## Conclusion

In conclusion, there is no evidence that lower limb muscle strength decreases during pregnancy. In contrast, we established a pregnancy induced change in the muscle architecture of the lower limb muscles which is likely to be caused by multiple factors such as changes in the endocrine system, body mass, and body composition. However, the risk of injury may be increased during pregnancy since muscle strength relative to body mass dropped in late pregnancy while the physical demand increases due to the increased body mass. Thus, strength exercises of the peripheral skeletal muscles and physical activity may benefit health during pregnancy.

## Ethics Statement

This study was carried out in accordance with the recommendations of the “Ethics Committee Charité – Universitätsmedizin Berlin” with written informed consent from all subjects. All subjects gave written informed consent in accordance with the Declaration of Helsinki. The protocol (No.: EA2/130/15) was approved by the “Ethics Committee Charité – Universitätsmedizin Berlin.”

## Author Contributions

KL conceived the design of the study. MB carried out the measurements and data analysis and drafted the manuscript. RM participated in the data collection and developed the data analysis algorithm. LH attended the measurements and was responsible for potential medical issues. KL and AA made important contributions during preparation and revision. All authors gave final approval for publication and agreed to be responsible for the content of the article.

## Conflict of Interest Statement

The authors declare that the research was conducted in the absence of any commercial or financial relationships that could be construed as a potential conflict of interest.
